# Key Brainstem Structures Activated during Hypoxic Exposure in One-day-old Mice Highlight Characteristics for Modeling Breathing Network in Premature Infants

**DOI:** 10.3389/fphys.2016.00609

**Published:** 2016-12-09

**Authors:** Fanny Joubert, Camille Loiseau, Anne-Sophie Perrin-Terrin, Florence Cayetanot, Alain Frugière, Nicolas Voituron, Laurence Bodineau

**Affiliations:** ^1^Sorbonne Universités, UPMC Univ Paris 06, Institut National de la Santé et de la Recherche Médicale, UMR_S1158 Neurophysiologie Respiratoire Expérimentale et CliniqueParis, France; ^2^Sorbonne Paris Cité, Université Paris 13, EA2363 Hypoxie et PoumonsBobigny, France; ^3^Institut de Neurosciences de la Timone, Aix Marseille Université, Centre National de la Recherche Scientifique, UMR 7289Marseille, France

**Keywords:** c-FOS, catecholamine, hypoxic ventilatory depression, serotonin, PHOX2B, subcoeruleus nucleus

## Abstract

We mapped and characterized changes in the activity of brainstem cell groups under hypoxia in one-day-old newborn mice, an animal model in which the central nervous system at birth is particularly immature. The classical biphasic respiratory response characterized by transient hyperventilation, followed by severe ventilation decline, was associated with increased c-FOS immunoreactivity in brainstem cell groups: the nucleus of the solitary tract, ventral reticular nucleus of the medulla, retrotrapezoid/parafacial region, parapyramidal group, *raphe magnus* nucleus, lateral, and medial parabrachial nucleus, and dorsal subcoeruleus nucleus. In contrast, the hypoglossal nucleus displayed decreased c-FOS immunoreactivity. There were fewer or no activated catecholaminergic cells activated in the medulla oblongata, whereas ~45% of the c-FOS-positive cells in the dorsal subcoeruleus were co-labeled. Approximately 30% of the c-FOS-positive cells in the parapyramidal group were serotoninergic, whereas only a small portion were labeled for serotonin in the raphe *magnus nucleus*. None of the c-FOS-positive cells in the retrotrapezoid/parafacial region were co-labeled for PHOX2B. Thus, the hypoxia-activated brainstem neuronal network of one-day-old mice is characterized by (*i*) the activation of catecholaminergic cells of the dorsal subcoeruleus nucleus, a structure implicated in the strong depressive pontine influence previously reported in the fetus but not in newborns, (*ii*) the weak activation of catecholaminergic cells of the ventral reticular nucleus of the medulla, an area involved in hypoxic hyperventilation, and (*iii*) the absence of PHOX2B-positive cells activated in the retrotrapezoid/parafacial region. Based on these results, one-day-old mice could highlight characteristics for modeling the breathing network of premature infants.

## Introduction

Human infants, particularly premature infants, display frequent episodes of apnea, and bradypnea, and thus very common episodes of hypoxia during the postnatal period (Bryan et al., 1986; Carroll and Agarwal, 2010; Teppema and Dahan, 2010; Mathew, 2011). In newborn mammals, hypoxia elicits a biphasic respiratory response, characterized by a transient increase followed by a severe decline of ventilation called the hypoxic ventilatory depression (HVD; Neubauer et al., 1990; Gozal and Gaultier, 2001; Carroll and Agarwal, 2010; Teppema and Dahan, 2010). The hypoxemia resulting from HVD, especially when frequently repeated, may negatively affect cardiovascular and neurocognitive functions, neurocognitive outcome, and long-term quality of life. Dysfunction of the hypoxic ventilatory response (HVR) is suspected in respiratory diseases such as Sudden Infant Death Syndrome (SIDS). SIDS victims typically experience a centrally mediated life-threatening apnea, possibly related to exaggerated HVD, due to the activation of a defense mechanism of the fetus that limits its O_2_ consumption by stopping the respiratory activity *in utero* under hypoxemia (Poets et al., 1999; Hunt, 2001; Kinney et al., 2011; Lavezzi, 2015).

The magnitude of the initial hyperventilation in most mammalian species increases with maturation, whereas the magnitude of the late decline is greatest in newborns and decreases with maturity (Carroll and Agarwal, 2010; Teppema and Dahan, 2010). Preterm infants with a birth weight <1500 g, exhibit no initial hyperventilation (Alvaro et al., 1992; Mathew, 2011). Hyperventilation mainly results from the activation of structures of the medulla oblongata by excitatory inputs coming from peripheral chemoreceptors (Vizek et al., 1987; Finley and Katz, 1992; Waldrop and Porter, 1995; Blessing et al., 1999; Carroll and Agarwal, 2010; Teppema and Dahan, 2010): cells of the commissural and medial parts of the nucleus of the solitary tract (cNTS and mNTS) and the ventral reticular nucleus of the medulla (VLM), many of which are catecholaminergic (Erickson and Millhorn, 1991, 1994; Hirooka et al., 1997). Second order projections transmit the message to other neuronal populations, such as the CO_2_-activated neurons of the retrotrapezoid/parafacial respiratory group region (RTN/pFRG) shown to be PHOX2B-positive (Takakura et al., 2006; Guyenet and Bayliss, 2015), and neurons of the lateral parabrachial nucleus (lPB; Hayward and Felder, 1995). Central mechanisms contribute to a decline in ventilation in parallel with peripheral chemoreceptor activation; if the initial hyperventilation fails to restore sufficient PO_2_ in arterial blood, the central mechanisms reduce the metabolic demand of the respiratory musculature by lowering ventilation (Neubauer et al., 1990; Carroll and Agarwal, 2010; Teppema and Dahan, 2010). Various mechanisms underlying the hypoxic decline in ventilation have been proposed including: (*i*) hyperperfusion of medullary CO_2_-sensitive areas (Neubauer et al., 1990); (*ii*) hypometabolism leading to a drop in CO_2_ production (Mortola, 2004); (*iii*) release of neuromodulators such as adenosine (Runold et al., 1989; Neubauer et al., 1990; Kawai et al., 1995) or serotonin (5-HT) (Herman et al., 1999; Richter et al., 1999); and (*iv*) intrinsic activation of cells in the medulla oblonga by low PO_2_ (Nolan and Waldrop, 1993; Bodineau et al., 2001; Voituron et al., 2006, 2011).

Here, we examined hypoxia-induced changes in the activity of brainstem neuronal populations in one-day-old mice to characterize the neuronal brainstem component of the HVR encountered under hypoxia in newborn mammals, in particular in premature mammals. Our working hypothesis was that one-day-old mice constitute a pertinent model because their central nervous system at birth is immature relative to other newborn mammals, such as humans and rats, with respect to their neuroanatomy, neurogenesis, gliogenesis, myelinisation, and molecular and biochemical dynamics in telencephalic regions (Teppema and Dahan, 2010; Darnall et al., 2016; Mallard and Vexler, 2015). Cats and rats have been used for many years in studies to identify the underlying mechanisms of the hypoxic ventilatory response, but much work has focused on mice over the past decade because of the increasing availability of genetic mouse models (Gaultier et al., 2003). Cell populations involved in the HVR of mice, particularly at birth, are not fully known. Thus, we performed an expanded analysis of changes in *c-FOS* expression under hypoxia in brainstem areas related to respiratory control. Dual labeling allowed us to characterize various activated c-FOS-positive cell populations by assessing their catecholaminergic, serotoninergic, or PHOX2B immunoreactivites.

## Materials and methods

All experiments, approved by the Charles Darwin Ethics Committee for Animal Experimentation (Ce5/2011/05), were carried out in accordance with Directive 2010/63/EU of the European Parliament and of the Council of 22 September, 2010 and French law (2013/118). All efforts were made to minimize the number of animals used and their suffering. Animals were maintained on a 12-h light-dark cycle with free access to food and water.

Experiments were performed on 31 newborn C57Bl6J mice (24.6 ± 2.6 h old; Charles River Laboratories, l'Arbresle, France).

### The hypoxic respiratory response

#### Recording of respiratory variables

Changes in ventilatory variables were non-invasively measured during the apnea-free period using a home-made whole body plethysmograph (Bartlett and Tenney, 1970) on 12 newborn mice. Animals were placed in an experimental chamber (20 ml) in which they freely moved. The chamber was connected to a reference box of the same size equipped with a temperature sensor (Newport Electronic, Santa Ana, CA, USA) that permits temperature control. The temperature of the animals was measured before and after each experiment via an oral probe. During recording sessions, the chamber was sealed and air flow to the chamber interrupted for 20 s (2 and 25 min after the beginning of hypoxia), leading to a change in pressure because the volume of the chamber was fixed. Between two recording sessions, the chamber was ventilated either with a humidified normoxic mixture (21% O_2_, 79% N_2_) or a hypoxic mixture (11% O_2_, 89% N_2_) heated to a temperature of ~31°C, the thermoneutral zone (Gordon, 1993), using an external heat source. The pressure change induced by the respiratory flow was assessed by connecting a differential pressure transducer (Valydine DP 45, Northridge, CA, USA) using an adaptation of the previously described barometric method (Bartlett and Tenney, 1970). The pressure signal was digitized through a Spike 2 data analysis system (Cambridge Electronic Design, Cambridge, UK). Measurement of the ventilatory variables was made on this signal *i.e*., respiratory frequency (*f*_R_), tidal volume (V_T_), minute ventilation ($\dot{V}$_E_), and the number of apneas per min (a ventilatory pause longer than twice the duration of the preceding respiratory cycles; Matrot et al., 2005; Menuet et al., 2011). The V_T_ was calculated using Drorbaugh and Fenn's equation (Drorbaugh and Fenn, 1955).

#### Respiratory variables observation

Prior to each experimental session, newborn mice were exposed to normoxic conditions for 20 min to become habituated to the chamber. Recordings were made under normoxic conditions for a further 15 min to define the control values. During the test period, the chamber was either flushed with the normoxic mixture (control group; *n* = 4) or the hypoxic mixture (hypoxic group; *n* = 8). Use of the control group ensured that the long period of retention in the recording chamber did not induce changes in the respiratory variables. At the end of the test period, newborn mice were removed from the chamber and the buccal temperature was taken. The respiratory variables were measured during the apnea-free periods during the hypoxic test (2 and 25 min after the beginning of hypoxia) and expressed as the percentage of control values. For the V_T_ calculation under hypoxia, the buccal temperature used was that measured before mice were placed in the recording chamber for the value at 2 min of hypoxia and that measured immediately at the end of the test for the value at 25 min of hypoxia.

Each recording session lasted ~20 s; a calibration volume of 100 μl was injected into the plethysmographic chamber with a Hamilton syringe toward the end of each recording session. Each newborn mouse was exposed only one time to the protocol. Recordings were made during 20-s periods when the chamber was sealed, and not continuously. Values are presented as mean ± standard error of the mean (SEM). D'Agostino-Pearson omnibus normality test was realized to assess the distribution of the data. The hypoxic ventilatory response was evaluated by one-way ANOVA for repeated measures followed by a Tukey's multiple comparisons test (GraphPad Prism5 San Diego California USA). Differences were considered to be significant if *p* < 0.05.

### Analysis of hypoxia-responding brainstem areas

#### Induction of c-FOS

The detection of c-FOS requires minimizing manipulations that could induce changes of cell activity unrelated to the studied stimulus and a sufficiently long induction period to induce detectable changes in *c-fos* expression (Herdegen and Leah, 1998; Perrin-Terrin et al., 2016). The protocol used for inducing *c-FOS* expression was similar to that used for analyzing the effect of hypoxia on respiratory variables with two exceptions: (*i*) the duration of the test period was 90 min (to facilitate c-FOS induction as c-FOS protein has a half-life of 90–100 min; Herdegen and Leah, 1998) and (*ii*) the absence of a recovery period to exclude the possibility that changes of neuronal activity revealed by c-FOS protein detection might be related to post-hypoxic neuronal mechanisms (Morris et al., 2003). At the end of the hypoxic period, newborn mice were placed under deep cold anesthesia (Danneman and Mandrell, 1997) and the brainstems removed.

#### Immunohistological procedures

Immunohistochemical analysis for c-FOS was carried out in mouse brainstems exposed to either normoxia or hypoxia (*n* = 19) to identify hypoxia-induced changes in cell activity. Brainstems were fixed in 4% paraformaldehyde in 0.1 M phosphate buffer (pH 7.4) for 48 h at 4°C (Voituron et al., 2011). They were then cryoprotected for 48 h in 30% sucrose in 0.1 M PBS and stored at −20°C for subsequent use. Standard immunohistochemical procedures were used to locate c-FOS on 40 μm-thick coronal free-floating sections obtained using a cryostat (Leica CM 1510S; Bodineau et al., 2011; Voituron et al., 2011).

The detection of c-FOS was coupled with that of tyrosine hydroxylase (TH), 5-HT, or PHOX2B to characterize the cells displaying changes in activity revealed by c-FOS analysis.

The manufacturer verified the specificity of primary antibodies in all cases and in addition, control sections were processed in parallel, but with the omission of the primary or secondary antibodies; we observed no labeling in the absence of the primary or secondary antibodies.

#### c-FOS immunohistochemistry

Sections were incubated with a rabbit polyclonal antibody against c-FOS (sc-52; Santa Cruz Biotechnology Inc., Santa Cruz, CA, USA; 1:2000) for 48 h at 4°C. They were then incubated for 2 h with a biotinylated goat anti-rabbit immunoglobulin (Vector Laboratories, Burlington, Canada; 1:500) followed by an avidin-biotin-peroxidase complex (Novostain Super ABC kit, Novocastra Laboratories, Newcastle, UK; 1:250) for 1 h. Peroxidase activity was detected using 0.02% 3,3′-diaminobenzidine tetrahydrochloride, 0.04% nickel ammonium sulfate, and 0.01% H_2_O_2_ in 0.05 M Tris-HCl buffer (pH 7.6).

Sections were mounted in sequential caudo-rostral order on silane-treated slides, air-dried, dehydrated with absolute alcohol, cleared with xylene, and coverslipped using Depex.

#### Dual immunohistochemistry for c-FOS/TH and c-FOS/5-HT

c-FOS was detected using a rabbit polyclonal antibody against c-FOS (sc-253 Santa Cruz Biotechnology Inc., Santa Cruz, CA, USA; 1:8000; 48 h; 4°C) according to the same protocol as above. The free-floating sections were then incubated with either a mouse polyclonal antibody against TH (MAB318, Millipore, 1:4000) or a rabbit polyclonal antibody against 5-HT (S5545, Sigma–Aldrich, Saint-Quentin Fallavier, France; 1:500; 48 h; 4°C). Sections were subsequently incubated for 2 h with biotinylated horse anti-mouse (Vector Laboratories, Burlington, Canada; 1:500) or goat anti-rabbit (Vector Laboratories, Burlington, Canada; 1:500) antibodies, and then with an avidin-biotin-peroxidase complex (1:250). The TH and 5-HT immunoreactivities were detected by incubation for 3–5 min in NovaRED (Vector Laboratories, Burlington, Canada).

Sections were mounted in sequential caudo-rostral order on silane-treated slides as for the single immunohistochemical detection of c-FOS. They were air-dried, dehydrated with absolute alcohol, cleared with xylene, and coverslipped using Entellan® (VWR International S.A.S).

#### Dual immunohistofluorescence for c-FOS/PHOX2B

Sections were first incubated with a primary antibody against PHOX2B (sc-13226, Santa Cruz biotechnology INC, Santa Cruz, CA, USA; 1:500; 48 h, 4°C), then with an Alexa 488-labeled donkey anti-goat antibody (Molecular Probes, Eugene, OR, 2 h at room temperature). Sections were then incubated with a c-FOS rabbit polyclonal antibody against the c-FOS protein (sc-253 Santa Cruz Biotechnology Inc., Santa Cruz, CA, USA; 1:4000; 48 h; 4°C), and then with an Alexa 555-labeled goat anti-rabbit antibody concomitantly with DAPI at 1:4000 (Molecular Probes, Eugene, OR, 2 h at room temperature). Sections were then washed, mounted in sequential caudo-rostral order on silane-treated slides, air-dried, and coverslipped using AquaPolyMount (Biovalley, Marne La Vallée, France).

#### Quantitative analysis of the effect of hypoxia on the number of c-FOS-positive cells and their characterization

Sections were examined under a light and fluorescence microscope (Leica DM 2000, Leica Microsystems, Heidelberg, Germany). We analyzed c-FOS-positive cells in brainstem structures related to respiratory control: the A5 region (A5), lPB and medial parabrachial nucleus (mPB), Kölliker-Fuse nucleus (KF), locus coeruleus nucleus (LC), subcoeruleus nucleus (SubC; the dorsal and ventral parts were separated at the level of the ventral boundary of the trigeminal motor nucleus *i.e*., dSubC and vSubC) at the pontine level and the VLM, hypoglossal nucleus (12N), dorsal motor nucleus of vagus (10N), cNTS, mNTS, and ventrolateral part of the nucleus of the solitary tract (vlNTS), *raphe magnus, obscurus*, and *pallidus* nuclei (RMg, ROb, and RPa), facial nucleus (n7), and the ventral medullary surface. Definitions of boundaries of these structures were made according to the mouse brain atlas (Paxinos and Franklin, 2001; Paxinos et al., 2007). The VLM is a neuronal column ventral to the *ambiguus* nucleus including the A1C1 group of neurons and extending from the pyramidal decussation to the caudal edge of the facial nucleus. We made a distinction between the caudal part of the VLM (cVLM; from the pyramidal decussation to the caudal edge of the lateral paragigantocellulaire nucleus) and the rostral part of the VLM (rVLM; from the caudal edge of the lateral paragigantocellulaire nucleus to the caudal edge of the facial nucleus) using standard landmarks as previously described (Voituron et al., 2011). The pre-Bötzinger complex is located in the caudal part of the rVLM. We also distinguished near the ventral surface of the medulla: (*i*) the lateral and medial part of the RTN/pFRG *i.e*., the lateral RTN/pFRG and medial RTN/pFRG and (*ii*) a more medial area at the lateral edge of the pyramidal tract, the parapyramidal group (PP) (Berquin et al., 2000a; Stornetta et al., 2005; Voituron et al., 2011), based on previously published data (Voituron et al., 2006, 2011; Huckstepp et al., 2015). We localized all of these structures with the aid of numerous ventral, dorsal, and lateral landmarks (such as those indicated in **Figures 2–5**) to delimit the entire volume of each structure.

The distribution of c-FOS cells was plotted onto drawings with the aid of a drawing tube attached to the microscope to illustrate their distribution (**Figure 2**). c-FOS and double-labeled cells were also photographed with a digital camera (Leica DFC450C, Leica Microsystems, Heidelberg, Germany). C-FOS counts were performed by eye using a counting grid in the eyepiece of the microscope at x400 to count all immunolabeled cells by varying the micrometer of the microscope, which was essential for tissue sections of 40 μm. For dual labeling, the counts were performed either by eye (at x400 except for the locus coeruleus, the pallidus, and obscurus raphe nuclei where x1000 magnification was used due to the high density of TH and 5-HT in labeled cells) or using images obtained with a digital camera (c-FOS/PHOX2B). We compared the total number of cells under normoxia and hypoxia for each analyzed area (Table 1). We analyzed the differences between the mean numbers of neurons obtained under normoxia or hypoxia using GraphPad (GraphPad Prism5 San Diego California USA) and used the Mann-Whitney tests to determine significance. Differences were considered to be significant if *p* < 0.05.

**Table 1:** **Average number of C-FOS-positive cells in brainstem respiratory areas**.

	**Normoxia**	**Hypoxia**
**MEDULLA**		
cNTS	16.0 ± 1.7	39.7 ± 8.0^*^
		*P* < 0.033
mNTS	35.4 ± 6.4	139.8 ± 17.8^**^
		*P* < 0.008
vlNTS	42.8 ± 9.6	130.5 ± 19.9^*^
		*P* < 0.011
ROb	153.4 ± 11.7	87.6 ± 21.1
		ns
RPa	107.2 ± 31.6	180.1 ± 44.0
		ns
RMg	21.0 ± 2.0	50.2 ± 6.8^*^
		*P* < 0.023
rVLM	38.2 ± 9.7	66.0 ± 16.7
		ns
cVLM	18.6 ± 5.4	81.9 ± 16.4^**^
		*P* < 0.002
PP	19.4 ± 7.9	80.6 ± 20.9^*^
		*P* < 0.02
RTN/pFRG	12.5 ± 2.1	30.7 ± 7.2^*^
		*P* < 0.03
7N	275 ± 8.0	216.6 ± 43.8
		ns
10N	6.7 ± 2.4	23.6 ± 5.7
		ns
12N	182.1 ± 25.5	122.2 ± 11.31^*^
		*P* < 0.033
**PONS**		
A5	21.5 ± 6.6	30.3 ± 6.14
		ns
LC	37 ± 7.9	41 ± 9.4
		ns
dSubC	42.6 ± 9.1	170.1 ± 32.3^*^
		*P* < 0.048
vSubC	26.4 ± 11.2	56.6 ± 8.5
		ns
mPB	7.4 ± 1.4	46.0 ± 12.7^**^
		*P* < 0.008
lPB	6.8 ± 2.1	27.4 ± 6.35 ^*^
		*P* < 0.037
KF	7.2 ± 2.9	15.8 ± 6.1
		ns

*Values presented are total number of c-FOS-positive cells ± S.E.M*.

* *P < 0.05, hypoxic values relative to normoxic values*.

** *P < 0.01, hypoxic values relative to normoxic values. ns, not significant*.

*cNTS, commissural part of nucleus of the tractus solitarius; cVLM, caudal part of the ventrolateral reticular nucleus of the medulla; dSubC, dorsal part of the subcoeruleus nucleus; KF, Kölliker fuse nucleus; LC, locus coeruleus; lPB, lateral part of the parabrachial nucleus; mNTS, medial part of nucleus of the tractus solitarius; mPB, medial part of the parabrachial nucleus; PP, parapyramidal group; RMg, nucleus of the raphe magnus; RPa, nucleus of the raphe pallidus; ROb, nucleus of the raphe obscurus; rVLM, rostral part of the ventrolateral reticular nucleus of the medulla; RTN/pFRG, retrotrapezoid/parafacial region; vlNTS, ventrolateral part of nucleus of the solitary tract; vSubC, ventral part of the subcoeruleus nucleus*.

## Results

The mean age of the pups assessed in the study (*n* = 31) was 24.6 ± 2.6 h and the mean weight 1.42 ± 0.02 g. Their mean *f*_R_ was 139.0 ± 14.4 breaths·min^−1^, mean V_T_ 25.6 ± 7.6 μl·g^−1^, and mean $\dot{V}$_E_ 3.8 ± 1.0 ml·g^−1^·min^−1^ (*n* = 12).

### Hypoxia induced the classical biphasic respiratory response observed in newborns: an early and transient increase followed by a severe decline in ventilation

The respiratory variables of unrestrained one-day-old mice maintained in the recording chamber under normoxia remained stable throughout the recording period (*n* = 4). Baseline values were defined after 20 min of adaptation in the chamber and the following values of the respiratory variables expressed as the percentage baseline. At the end of the recording period, the mean *f*_R_ and mean V_T_ were 106.9 ± 14.3% and 120.9 ± 7.6% of control values, respectively, leading to a mean $\dot{V}$_E_ of 131.2 ± 12.4% of control values. This observation shows the stability of the respiratory variables during the period of retention in the recording chamber.

**Figure 1:**
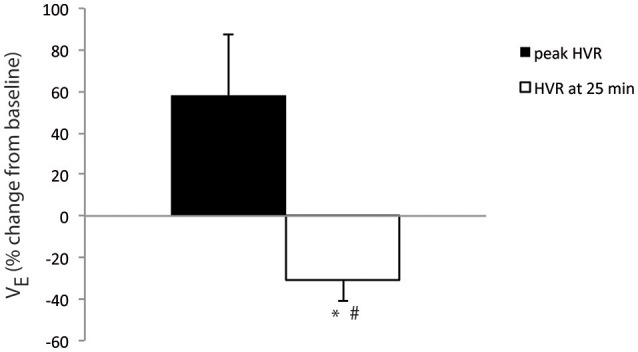
**One-day-old mice displayed a ventilatory depression in response to hypoxia**. Average percent change in $\dot{V}$_E_ from baseline at 2 min (peak HVR, black bar) and 25 min (white bar) of hypoxic exposure in one-day-old mice (*n* = 8). ^*^p < 0.05 *vs*. baseline, ^#^*p* < 0.05 *vs*. peak HVR.

The mean *f*_R_ was 166.1 ± 12.5 breaths·min^−1^, the mean V_*T*_ 35.6 ± 9.6 μl·g^−1^, and the mean $\dot{V}$_E_ 5.4 ± 1.2 ml·g^−1^·min^−1^ during the normoxic control period in the unrestrained one-day-old mice that were subsequently submitted to hypoxia (*n* = 8). The mean $\dot{V}$_E_ tended to peak at 158.1 ± 29.4% of control values 2 min after the onset of hypoxic exposure (Figure 1). It then decreased significantly dropping below control (*p* < 0.04) and below the high values observed at the onset of hypoxia (*p* < 0.03) to 68.9 ± 10.0% of control values 25 min after the onset of hypoxic exposure (Figure 1). Changes in $\dot{V}$_E_ were related to the respective effects of hypoxia on both *f*_R_ and V_T_. The mean *f*_R_ was at 115.4 ± 19.0% of control values 2 min after the onset of hypoxic exposure and at 86.5 ± 7.5% of control values 25 min after the onset of the hypoxic test. The mean V_T_ was at 138.8 ± 18.6% of control values 2 min after the onset of hypoxic exposure and at 73.7 ± 15.8% of control values 25 min after the onset of hypoxic exposure.

### Brainstem areas that participate in respiratory control have a low number of c-FOS-positive cells under normoxic conditions

**Figure 2:**
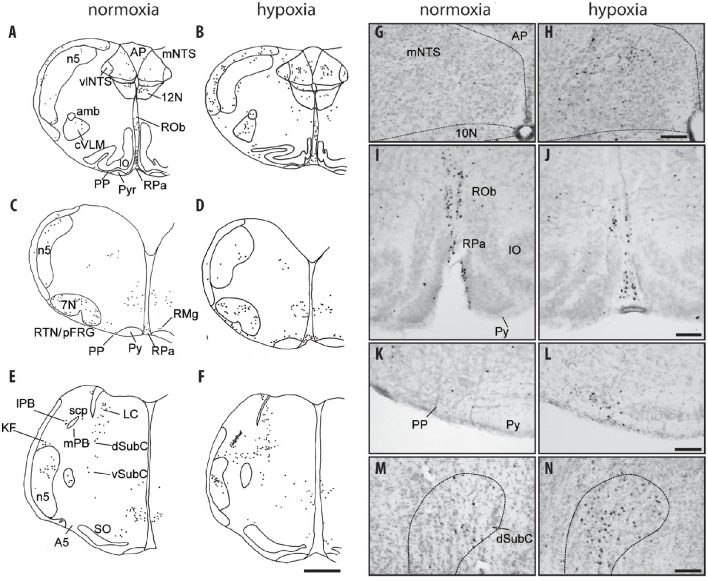
**c-FOS-positive cells in the medulla oblongata and pons of one-day-old mice under hypoxia**. Drawings of representative sections from the medulla oblongata **(A–D)** and pons **(E,F)** under normoxia **(A,C,E)** and hypoxia **(B,D,F)**. Scale bar = 500 μm. Photomicrographs of c-FOS immunoreactivity in the mNTS **(G,H)**, the RPa and ROb **(I,J)**, the PP **(K,L)**, and the dSubC **(M,N)** under normoxia **(G,I,K,M)** and hypoxia **(H,J,L,N)**. Scale bar = 100 μm. 7N, facial nucleus; 10N, dorsal motor nucleus of the vagus 12N, hypoglossal nucleus; A5, A5 region; Amb: ambiguus nucleus; AP, area postrema; dSubC, dorsal part of the subcoeruleus nucleus; mNTS, median part of the nucleus of the tractus solitarius; cVLM, caudal part of the ventrolateral medullary reticular nucleus; PP, parapyramidal group; Py, pyramidal tract; RPa, *raphe pallidus* nucleus; RMg, *raphe magnus* nucleus; RTN/pFRG, retrotrapezoid nucleus/parafacial respiratory group; vlNTS, ventrolateral part of the nucleus of the tractus solitarius; vSubC, ventral part of the subcoeruleus nucleus.

One-day-old mice maintained under normoxic conditions (control animals) had a relatively small number of c-FOS-positive cells both in respiratory related areas of the medulla oblongata: NTS, VLM, RTN/pFRG, PP, 10N, 12N, and medullary raphe nuclei (Table 1; Figures 2A,C,G,I,K), and related respiratory areas of the pons:. PB, KF, A5, LC, and SubC (Table 1; Figures 2E,M).

### Hypoxia induces an increase in c-FOS expression in brainstem areas that participate in respiratory control

#### Medulla oblongata

##### Nucleus of the solitary tract

**Figure 3:**
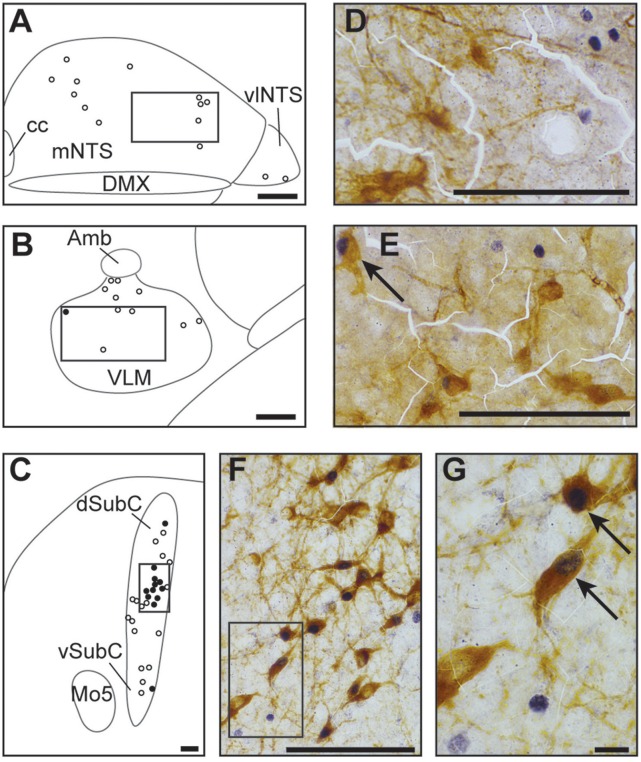
**Catecholaminergic character of hypoxic c-FOS-positive cells of one-day-old mice**. Drawings illustrating the distribution of cells immunoreactive for c-FOS (white points) or both c-FOS and TH (black points) in the mNTS, vlNTS **(A)**, VLM **(B)**, and dSubC and vSubC **(C)** under hypoxic conditions. Scale bar = 100 μm. Photomicrographs of sections double-immunolabeled for c-FOS (gray) and TH (brown) in the mNTS **(D)**, VLM **(E)**, and dSubC **(F)** corresponding to the regions outlined by the black rectangles in **(A**–**C)**, respectively. Scale bar = 100 μm. **(G)** photomicrograph representing an enlargement of the black rectangle in **(F)** Scale bar = 10 μm. Black arrows indicate c-FOS-positive neurons that are also immunoreactive with TH. Amb: ambiguus nucleus; cc: central canal; DMX, dorsal motor nucleus of vagus; dSubC, dorsal part of the subcoeruleus nucleus; mNTS, median part of the nucleus of the tractus solitarius; Mo5, motor trigeminal nucleus; VLM, ventrolateral medullary reticular nucleus; vlNTS, ventrolateral part of the nucleus of the tractus solitarius; vSubC, ventral part of the subcoeruleus nucleus.

The three analyzed subdivisions of the NTS *i.e*., cNTS, mNTS, and vlNTS, especially the mNTS, had significantly more c-FOS positive cells under hypoxic than normoxic conditions (Table 1; Figures 2B,H; +148, +235, and +205%, respectively). Virtually none of the c-FOS-positive cells were immunolabeled for TH (Figures 3A,D); we observed no dually labeled cells in the cNTS and vlNTS and only 0.3% in the mNTS. In addition, some of the c-FOS-positive cells of the cNTS and mNTS were also immunoreactive for PHOX2B: 18 and 25%, respectively.

##### Hypoglossal nucleus

There were slightly, but significantly, fewer c-FOS-positive cells in the 12N under hypoxia than normoxia (Table 1; Figure 2B; −33%).

##### Dorsal motor nucleus of vagus

We observed a higher of number of c-FOS-positive cells in the 10N under hypoxia than normoxia, but the difference was not significant (Table 1).

##### Ventrolateral reticular nucleus of the medulla

The cVLM, but not the rVLM, which encompasses the pre-Bötzinger complex, had more c-FOS-positive cells under hypoxic than normoxic conditions (Table 1; Figures 2A,B; +340%). Only a few of the hypoxic-c-FOS-positive cells of the cVLM were also immunoreactive for TH (Figures 3B,E; 5%).

##### Retrotrapezoid/parafacial region

**Figure 4:**
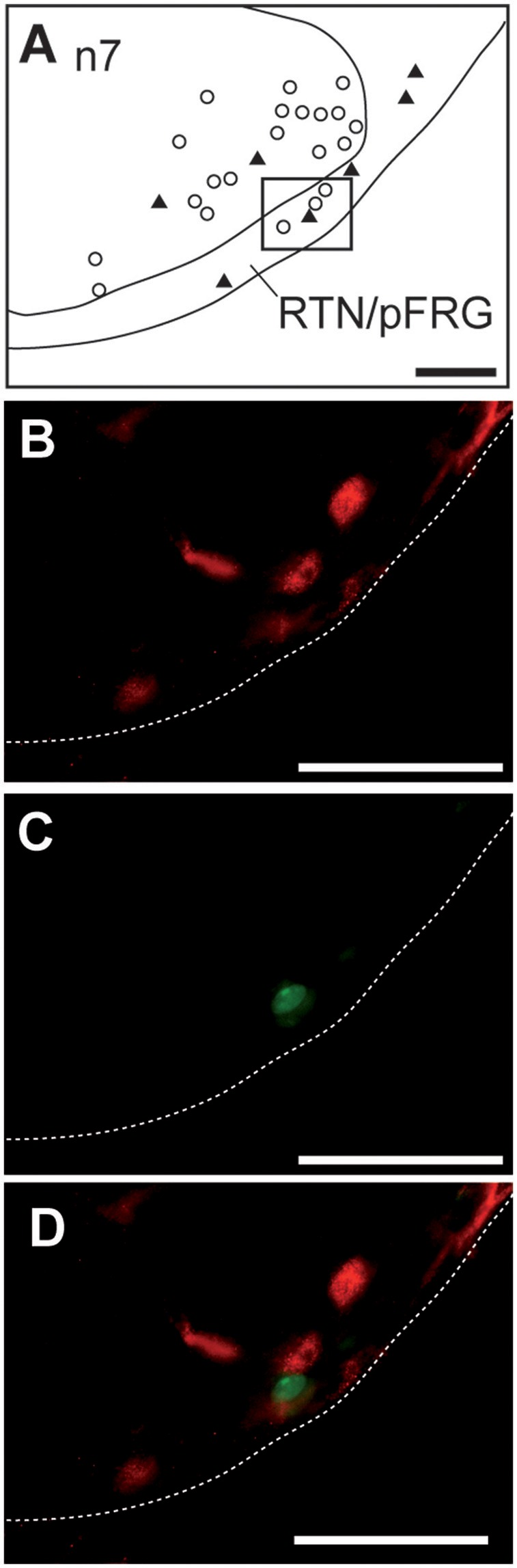
**Hypoxic c-FOS-positive cells of the RTN/pFRG in one-day-old mice are not immunolabeled for PHOX2B**. Drawing illustrating the distribution of cells immunoreactive for c-FOS and PHOX2B in the RTN/pFRG **(A)**. Scale bar = 100 μm. Solid black triangles indicate neurons that express PHOX2B and white points indicate c-FOS-positive neurons. Photomicrographs of a section double-immunolabeled for c-FOS (in red, **B,D**) and PHOX2B (in green, **C,D**) in the RTN/pFRG corresponding to the region outlined by the black rectangle **(A)**. Scale bar = 50 μm. n7, facial nucleus; RTN/pFRG, retrotrapezoid nucleus/parafacial respiratory group.

The RTN/pFRG region also had significantly more c-FOS-positive cells (~146%) under hypoxia than normoxia (Table 1; Figures 3C,D). None of the c-FOS-positive cells of the RTN/pFRG region were immunolabeled for PHOX2B (Figure 4).

##### Parapyramidal group

**Figure 5:**
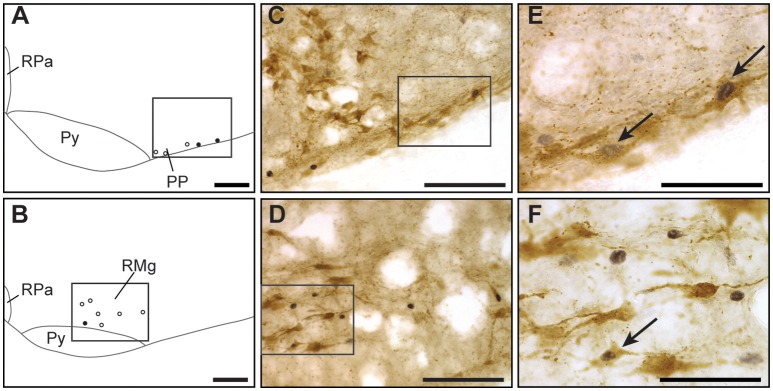
**Serotoninergic character of hypoxic c-FOS-positive cells of one-day-old mice**. Drawings illustrating the distribution of cells immunoreactive for c-FOS (white points) or both c-FOS and 5-HT (black points) in the PP **(A)** and RMg **(B)** under hypoxic conditions. Scale bar = 100 μm. Photomicrographs of sections double-immunolabeled for c-FOS (gray) and 5-HT (brown) in the PP **(C)** and RMg **(D)** corresponding to the regions outlined by the black rectangles of **(A,B)**, respectively. Scale bar = 100 μm. **(E,F)** photomicrographs representing an enlargement of the black rectangles in **(C,D)**, respectively. Scale bar = 50 μm. Black arrows indicate c-FOS-positive neurons that are also immunoreactive for 5-HT. RPa, *raphe pallidus* nucleus; RMg, *raphe magnus* nucleus; PP, parapyramidal group; Py, pyramidal tract.

The group of cells adjacent to the RTN/pFRG, called the PP, also had more c-FOS-positive cells under hypoxia than normoxia (Table 1; Figures 2A–D,K,F; +315%). A substantial portion of these cells, 30%, were also immunoreactive for 5-HT (Figures 5A,C,E).

##### Medullary raphe nuclei

Among medullary *raphe* nuclei, the RMg displayed more c-FOS-positive cells under hypoxic than normoxic conditions (Table 1; Figures 2C,D; +139%). Only a small portion of RMg hypoxic-c-FOS-positive cells, 15%, were also immunoreactive for 5-HT (Figures 5B,D,F). In contrast, there were fewer c-FOS-positive cells under hypoxia than normoxia in the ROb but the difference was not significant (Table 1; Figures 2–D,I,J; −43%) and no change in the number of c-FOS-positive cells in the RPa (Table 1; Figures 2B,J).

#### Pons

##### Parabrachial and Kölliker fuse nuclei

In the PB complex, both the lPB and mPB showed substantially more c-FOS-positive cells under hypoxia than normoxia (Table 1; Figures 2E,F; +303 and +522%, respectively). In contrast, there was no significant difference in the number of c-FOS-positive cells in the KF under hypoxia (Table 1; Figures 2E,F).

##### Locus coeruleus and subcoeruleus nucleus

There were significantly more c-FOS-positive cells in the dSubC under hypoxic conditions (Table 1; Figures 2E,F,M,N; +299%) but not in the vSubC (Table 1; Figures 2E,F). Among the hypoxic-c-FOS-positive cells of the dSubC, 45% were also immunoreactive for TH (Figures 3C,F,G). Additionally, there was no difference in the number of c-FOS-positive cells in the LC (Table 1; Figures 2E,F).

##### A5 region

We observed no difference in the number of c-FOS-positive cells in A5 between hypoxic and normoxic conditions (Table 1; Figures 2E,F).

## Discussion

We described and characterized the brainstem neuronal network activated by hypoxia in one-day-old mice by analyzing c-FOS protein levels by immunohistochemistry. Our main finding is that one-day-old mice displayed activation of catecholaminergic cells of the dSubC, an area implicated in the strong depressive pontine influence under hypoxia previously reported in the fetus, but not in newborns or adults of other mammalian species (Breen et al., 1997; Teppema et al., 1997; Berquin et al., 2000a,b; Bodineau and Larnicol, 2001). Our results also revealed that the hypoxia-activated brainstem neuronal network of one-day-old mice is characterized by weak or absent activation of cells in areas involved in hypoxic hyperventilation *i.e*., catecholaminergic neurons of the VLM and PHOX2B cells of the retrotrapezoid/parafacial region (Erickson and Millhorn, 1991, 1994; Takakura et al., 2006) as suggested by the scarcity or absence of TH-positive or PHOX2B-positive cells among the c-FOS-positive-cells.

### Whole body flow barometric plethysmography in one-day-old mice

We measured respiratory variables using whole-body flow barometric plethysmography in freely moving newborn mice to limit confinement-related stress. This is a commonly used technique to measure pulmonary ventilation in unrestrained and non-anesthetized animals that consists of recording the changes of pressure in a chamber caused by breathing (Tree et al., 2014). As the inspired gas is warmed and humidified from the ambient to the pulmonary values, the total pressure in the recording chamber increases; the opposite occurs during expiration. The accuracy of V_T_ calculations based on whole-body plethysmography data of small animals has been questioned (Enhorning et al., 1998; Mortola and Frappell, 1998). The body temperature, humidity, and temperature of the recording chamber are difficult to control due to the small size of the devices used for such small animals, leading to highly variable absolute V_T_ and $\dot{V}$_E_ values. These values should thus be viewed with caution. This has led researchers to base their analysis on the changing values of V_T_ and $\dot{V}$_E_ as a percentage of control values. Furthermore, small mammals, particularly newborns, develop hypoxic hypothermia due to hypometabolism and increased heat loss. In our experiments, the temperature of the animal was evaluated before and after each experiment via an oral probe because of the difficulty to measure body temperature continuously with our home-made plethysmograph. Thus, our plethysmographic data should be taken with caution. Indeed, the plethysmography was used primarily to simply assure the presence of a biphasic respiratory response in one day old mice, as classically described in other newborns (Simakajornboon et al., 2004; Bairam et al., 2013).

In addition to these considerations, the pups were isolated from their mother for a long time during the plethysmographic recordings. We observed no significant modification of the respiratory variables in animals maintained under normoxia despite this long separation that may have led to physiological changes. This indicates that changes in the respiratory variables developed by newborn mice submitted to hypoxic conditions are effectively due to the reduction in O_2_, and not their long retention.

### Neurons of the subcoeruleus nucleus, an area implicated in the strong depressive pontine influence under hypoxia in the fetus, are activated by hypoxia in one-day-old mice

Analysis of c-FOS protein revealed increased activity in neurons of the mPB and dSubC. Such an increase has not been reported in other newborn rodents (see the Figure 3 in Berquin et al., 2000b). In the fetus of sheep, the participation of cells located in the dorsolateral part of the pons to HVD, more precisely those in the mPB, KF, and SubC, has already been discussed in the literature (Gluckman and Johnston, 1987; Walker, 1995; Breen et al., 1997; Nitsos and Walker, 1999; Walker et al., 2000; Teppema and Dahan, 2010). Hypoxia induces an increase in *c-FOS* expression in neurons of the mPB and SubC in fetal, but not newborn, sheep (Breen et al., 1997; Nitsos and Walker, 1999). The hypoxia-activated cells of the SubC have been proposed to be O_2_ sensors and to strongly inhibit breathing in fetal sheep, but to lose this effect after birth because of their inhibition by peripheral chemoreceptors (Breen et al., 1997). The present observed increase in c-FOS-positive cells in the dSubC, of which a significant portion are catecholaminergic, may be indicative of activated cells with similar properties to those identified in the fetus by Walker and collaborators (Walker, 1995; Breen et al., 1997; Nitsos and Walker, 1999). In such a model, they may be involved in HVD in one-day-old mice and constitute a non-inhibited fetal mechanism at birth different from that of other studied newborn mammals, such as newborn rats, which are more mature at birth than mice (Gauda, 2006). This feature of one-day-old mice may make this model particularly suitable for mechanistic studies on the occurrence of excessive HVD in premature newborns or even on the occurrence of SIDS. Indeed, SIDS has been hypothesized to result from the awakening of a defense mechanism of the fetus consisting of a strong depressive response to hypoxemia that limits O_2_ consumption (Lavezzi, 2015). A recent publication by Lavezzi and collaborators emphasized hypoplasia and neurochemical alterations of KF neurons underlying the pathogenetic mechanisms of SIDS (Lavezzi, 2015). Our present data and previously published work in fetal and newborn sheep (Breen et al., 1997) underscore the interest in searching for possible alterations in the integrity of dSubC neurons in the brainstems of infants who died of SIDS.

In rats, SubC and mPB have been shown to contain pre-motoneurons that innervate hypoglossal motoneurons involved in maintaining upper airway patency during breathing (Dobbins and Feldman, 1995; Fay and Norgren, 1997). The presently observed decrease in c-FOS protein levels in the hypoglossal nucleus cells may be linked to an inhibitory drive coming from the dSubC and mPB during hypoxia. This hypothetic inhibitory pathway may contribute to HVD by reducing the airway opening when activated under hypoxia. Noradrenergic cells of the dSubC innervate the hypoglossal nucleus, of which the motoneurons are activated by noradrenalin (Aldes et al., 1992; Fenik et al., 2008; Funk et al., 2011). If dSubC cells are involved in the decrease in the expression of *c-FOS* in the hypoglossal nucleus, they would not be those immunoreactive for TH. Further experiments are necessary to determine the pharmacological phenotype of the c-FOS-positive, but TH-negative, cells of the dSubC.

### Hypoxia in one-day-old mice induces an increase in the number of c-FOS-positive cells in the nucleus *raphe magnus*, an area previously described as having a hypoxic respiratory depressive influence

We observed a large increase in the number of c-FOS-positive cells under hypoxia in the RMg. Such an increase has not been reported in newborns of other species (Breen et al., 1997; Teppema et al., 1997; Horn et al., 2000; Berquin et al., 2000b) or adults subjected to moderate hypoxia (Berquin et al., 2000a), but has been reported in adults under severe hypoxia (Erickson and Millhorn, 1994). Based on data from the literature, we suggest that hypoxia-activated RMg neurons contribute to HVD. Indeed, activation of RMg neurons attenuate the activation of NTS neurons by peripheral chemoreceptors (Perez and Ruiz, 1995) and RMg neurons exert a moderating influence on the V_*T*_ under hypoxic conditions (Gargaglioni et al., 2003). Thus, activation of RMg neurons in one-day-old mice may participate in the HVD. With the exception of a few cells, the hypoxic c-FOS-positive cells that we observed were not serotoninergic. In addition to serotoninergic neurons, the RMg contains GABAergic neurons that could be involved in HVD. This is supported by the fact that *iv* administration of bicuculline, a GABA_A_ antagonist, reduces the respiratory inhibition that occurs after electrical stimulation of the RMg (Cao et al., 2006).

### Hypoxia induces an increase in c-FOS expression in NTS and VLM, two structures that participate in hypoxic hyperventilation, but there is little or no c-FOS protein in catecholaminergic neurons

Hypoxia induced an increase in the number of c-FOS-positive cells in areas recognized to be involved in early hypoxic hyperventilation triggered by stimulation of peripheral chemoreceptors (*see for revue* Teppema and Dahan, 2010) *i.e*., the cNTS, mNTS, and lPB. The cNTS and mNTS constitute the major central site for the integration of inputs from peripheral chemoreceptors whereas only few afferents from peripheral chemoreceptors project toward the VLM (Finley and Katz, 1992). The two subnuclei of the NTS are the first relay between peripheral afferences and the VLM and lPB (Nunez-Abades et al., 1993; Hayward and Felder, 1995). All of these connections conceivably provide a common basis for the hypoxic increase in *c-FOS* expression that we observed in these structures in one-day-old mice and that has been observed in other species at several stages of development (Teppema et al., 1997; Berquin et al., 2000b).

The dual detection of c-FOS and TH revealed that no, or only very few, neurons activated by hypoxia in the cNTS, mNTS, and VLM were catecholaminergic. Previous data about the catecholaminergic character of c-FOS-positive hypoxia-activated cells in these structures made in adult mammals have showed that no, or very few, c-FOS hypoxia-activated neurons in the NTS are also catecholaminergic, whereas a large portion of c-FOS hypoxia-activated neurons in the VLM are also immunoreactive for TH (Erickson and Millhorn, 1994; Soulier et al., 1997; Teppema et al., 1997). The only data from the literature concerning this issue in newborn animals is the study of *c-FOS* expression in newborn rats under high-altitude conditions showing that an altitude of 8000 m induces an increase in the number of c-FOS positive cells in the NTS and VLM with a large portion of them in the VLM also immunoreactive for TH (Kaur et al., 2001). Taking into account these data, our results suggest that the effect of hypoxia on the VLM differed in one-day-old mice from other studied newborn or adult mammals. This difference may depend on the maturation state of either VLM neurons or brainstem connections. The absence of VLM catecholaminergic neuron activation in one-day-old mice could conceivably participate in HVD, and even strengthen it in this model, as they have been associated with hypoxic hyperventilation (Erickson and Millhorn, 1994; Soulier et al., 1997; Teppema et al., 1997). If this observation is likely due to the degree of immaturity of the central nervous system of the one-day-old mice as we assume (Teppema et al., 1997; Gauda, 2006; Mallard and Vexler, 2015), a similar mechanism may be involved in exacerbating the respiratory problems encountered at birth in premature infants.

### The ventral medullary surface displays increased c-FOS expression in one-day-old mice in serotonin- but not PHOX2B-positive neurons

We observed an increase in the number of c-FOS-positive cells in the RTN/pFRG. The RTN/pFRG is involved in the respiratory adaptation to both hypercapnia (Teppema et al., 1997; Nattie, 2001; Okada et al., 2002; Guyenet et al., 2005; Stornetta et al., 2006; Teppema and Dahan, 2010; Guyenet and Bayliss, 2015) and hypoxia (Bodineau et al., 2000a, 2001; Bodineau and Larnicol, 2001; Takakura et al., 2006; Voituron et al., 2006, 2011; Teppema and Dahan, 2010). The RTN/pFRG contains both CO_2_ and O_2_ sensor cells (Guyenet et al., 2005; Voituron et al., 2006, 2011; Onimaru et al., 2008; Lazarenko et al., 2009; Guyenet and Mulkey, 2010) and also integrates chemosensory inputs from peripheral chemoreceptors (Berquin et al., 2000a; Bodineau et al., 2000b; Takakura et al., 2006). The well-identified CO_2_-sensor cells of the RTN/pFRG (Lazarenko et al., 2009; Guyenet and Mulkey, 2010) are derived from neurons that express *Phox2b, Atoh-1*, and *Egr-2*, and are characterized by the presence of PHOX2B, NK1 receptors, VGLUT2, TASK-2, GPR4, and GALANIN (Weston et al., 2004; Stornetta et al., 2006; Onimaru et al., 2008; Dubreuil et al., 2009; Rose et al., 2009; Guyenet and Mulkey, 2010; Guyenet and Bayliss, 2015; Ruffault et al., 2015). In the present work, none of the c-FOS positive cells of the RTN/pFRG were PHOX2B-positive. This suggests that although the one-day-old mice displayed severe hypoventilation under hypoxia, hypoventilation may not entail an increase in CO_2_ that should have been detected by the PHOX2B cells of the RTN/pFRG. Regardless of the mechanisms responsible for HVD, the concomitant hypometabolism that maintains isocapnia, which constitutes a feature of the newborn response to hypoxia (Mortola, 2004), appears to be extremely effective in one-day-old mice. The activation of PHOX2B-negative RTN/pFRG cells may depend on intrinsic O_2_-sensing properties that we previously demonstrated by measuring *c-FOS* expression in brainstem spinal cord preparations from newborn rodents (Voituron et al., 2006, 2011). It is unlikely that the increase in the number of c-FOS-positive/PHOX2B-negative cells in RTN/pFRG depends on peripheral chemoreceptor inputs. Takakura and collaborators have shown that such indirect activation implicates cNTS glutamatergic neurons projecting to PHOX2B RTN/pFRG cells (Takakura et al., 2006). This suggests possible immaturity of the connection between cNTS cells activated by peripheral chemoreceptors and RTN/pFRG PHOX2B-positive cells.

We observed a greater increase in *c-FOS* expression in the more medial group, called PP, with 30% of the c-FOS-positive cells containing 5-HT, than for the RTN/pFRG. This group of cells has been suggested to be the positional homolog of the human medullary arcuate nucleus (Filiano and Kinney, 1992), a structure in which abnormalities have been reported in infants who died of SIDS (Filiano and Kinney, 1992; Paterson et al., 2006; Kinney et al., 2011). The increase in c-FOS protein levels induced by hypoxia in the PP is of particular interest given the possible involvement of both the arcuate nucleus and abnormalities in the respiratory response to hypoxia in SIDS (Paterson et al., 2006; Kinney et al., 2011). The PP contains GABAergic and serotoninergic neurons (Stornetta and Guyenet, 1999; Weston et al., 2004; Stornetta et al., 2005) and displays multiple sites of projections including the pre-Bötzinger complex and intermediolateral column (Holtman et al., 1990; Jansen et al., 1995). Functionally, the PP has been reported to be involved in autonomic regulation under hypoxia. Darnall and collaborators reported that the destruction of 5-HT medullary neurons, including those of the ROb and PP increase the arousal latency from sleep induced by hypoxia in newborn rats (Darnall et al., 2016). In the relatively immature one-day-old mouse (Gauda, 2006), we only observed an increase in c-FOS- and 5-HT-positive cells in the PP, but not in the ROb, where the number of c-FOS-positive cells tended to decrease. Thus, our results suggest that 5-HT PP neurons must have a critical role in arousal from sleep under hypoxia in immature newborn mammals. Our results combined with the fact that hypoplasia of this region has been shown in newborn death due to SIDS, suggests that the dysfunction of 5-HT neurons of the PP in infants, and particularly in premature infants, could result in a high risk situation due to the decrease of arousal from sleep during hypoxia.

## Conclusion

This study significantly contributes to the knowledge of key brainstem cell populations, for which the activity is modulated during hypoxia using an animal model characterized by its immaturity relative to other mammals, the one-day-old mouse (Gauda, 2006; Gaultier et al., 2006; Teppema and Dahan, 2010; Darnall et al., 2016; Mallard and Vexler, 2015). Our results highlight changes in the activity of cell populations that may participate in the respiratory depression of this animal model *i.e*., the activation of catecholaminergic cells of the dSubC, an area previously implicated in a strong depressive pontine influence in the fetus but not in newborns. Also, we did not observe an increase in the number of c-FOS-positive cells commonly associated with the development of hypoxic hyperventilation, catecholaminergic cells of VLM, and PHOX2B-postive neurons of the RTN/pFRG. This was suggested by the scarcity or absence of TH-positive or PHOX2B-positive cells among the c-FOS-positive-cells. Finally, our results suggest that 5-HT neurons of the PP, shown to be involved in arousal from sleep, are the only serotoninergic medullary neurons activated by hypoxia in immature mammals. Some physiopathological conditions in which depressant and arousing mechanisms would be more or less potent might lead to life-threatening situations, especially in premature infants. In conclusion, one-day-old mice highlight characteristics to model dysfunction of the breathing network in premature infants. In the absence of data in the literature, future experiments to explore *c-FOS* expression in older mice, displaying a mature hypoxic ventilatory response, would help to exclude the possibility that the pattern of *c-FOS* expression observed in one-day-old mice is simply species specific and not due to their relative immaturity.

## Author contributions

FJ, CL, and AP performed experiments, analyzed data, and generated the figures. FC performed experiments, analyzed data, and made comments on the manuscript. AF discussed the results and their significance and made comments on the manuscript. NV performed experiments, generated a figure, analyzed, and interpreted data, discussed the results and their significance, and made comments on the manuscript. LB designed and supervised all experiments, obtained funding, shaped, and interpreted the data, generated a figure, discussed the results, and their significance, and wrote the manuscript.

## Funding

This work received financial support from the “Legs Poix, Chancellerie des Universités de Paris” (Legs 1504) and the French Government-Institut Hospitalo-Universitaire-A-Institut du Cerveau et de la Moelle Epinière (IHU-A-ICM) “Investissement d'Avenir” ANR-10-IAIHU-06 program. FJ was supported by the “Fonds de Dotation pour la Recherche en Santé Respiratoire 2012.”

### Conflict of interest statement

The authors declare that the research was conducted in the absence of any commercial or financial relationships that could be construed as a potential conflict of interest.

## Abbreviations

5HT: serotonin
A5: A5 region
cNTS: commissural part of the nucleus of the solitary tract
cVLM: caudal part of the ventrolateral reticular nucleus of the medulla
dSubC: dorsal part of the subcoeruleus nucleus
*f*_R_: respiratory frequency
HVD: hypoxic ventilatory depression
HVR: hypoxic ventilatory response
KF: Kölliker-Fuse nucleus
LC: locus coeruleus nucleus
lPB: lateral parabrachial nucleus
mNTS: medial part of the nucleus of the solitary tract
mPB: medial parabrachial nucleus
n7: facial nucleus
PP: parapyramidal group
RMg: raphe magnus nucleus
ROb: raphe obscurus nucleus
RPa: raphe pallidus nucleus
RTN/pFRG: retrotrapezoid nucleus/parafacial respiratory group region
rVLM: rostral part of the ventrolateral reticular nucleus of the medulla
SIDS: Sudden Infant Death Syndrome
TH: tyrosine hydroxylase
$\overset{\cdot }{\text{V}}$_E_: ventilation minute
vlNTS: ventrolateral part of the nucleus of the solitary tract
vSubC: ventral part of the Subcoeruleus nucleus
V_T_: tidal volume.

## References

[B1] Aldes L. D. Chapman M. E. Chronister R. B. Haycock J. W. (1992). Sources of noradrenergic afferents to the hypoglossal nucleus in the rat. Brain Res. Bull. 29, 931–942. 10.1016/0361-9230(92)90168-W 1282080

[B2] Alvaro R. Alvarez J. Kwiatkowski K. Cates D. Rigatto H. (1992). Small preterm infants (less than or equal to 1500 g) have only a sustained decrease in ventilation in response to hypoxia. Pediatr. Res. 32, 403–406. 10.1203/00006450-199210000-00007 1437391

[B3] Bairam A. Lumbroso D. Joseph V. (2013). Effect of progesterone on respiratory response to moderate hypoxia and apnea frequency in developing rats. Respir. Physiol. Neurobiol. 185, 515–525. 10.1016/j.resp.2012.11.001 23153693

[B4] Bartlett D. Jr. Tenney S. M. (1970). Control of breathing in experimental anemia. Respir. Physiol. 10, 384–395. 10.1016/0034-5687(70)90056-3 5476156

[B5] Berquin P. Bodineau L. Gros F. Larnicol N. (2000a). Brainstem and hypothalamic areas involved in respiratory chemoreflexes: a Fos study in adult rats. Brain Res. 857, 30–40. 10.1016/S0006-8993(99)02304-5 10700550

[B6] Berquin P. Cayetanot F. Gros F. Larnicol N. (2000b). Postnatal changes in Fos-like immunoreactivity evoked by hypoxia in the rat brainstem and hypothalamus. Brain Res. 877, 149–159. 10.1016/S0006-8993(00)02632-9 10986327

[B7] Blessing W. W. Yu Y. Nalivaiko E. (1999). Medullary projections of rabbit carotid sinus nerve. Brain Res. 816, 405–410. 10.1016/S0006-8993(98)01147-0 9878853

[B8] Bodineau L. Larnicol N. (2001). Brainstem and hypothalamic areas activated by tissue hypoxia: Fos-like immunoreactivity induced by carbon monoxide inhalation in the rat. Neuroscience 108, 643–653. 10.1016/S0306-4522(01)00442-0 11738500

[B9] Bodineau L. Cayetanot F. Frugiere A. (2000a). Possible role of retrotrapezoid nucleus and parapyramidal area in the respiratory response to anoxia: an *in vitro* study in neonatal rat. Neurosci. Lett. 295, 67–69. 10.1016/S0304-3940(00)01590-1 11078938

[B10] Bodineau L. Cayetanot F. Frugiere A. (2001). Fos study of ponto-medullary areas involved in the *in vitro* hypoxic respiratory depression. Neuroreport 12, 3913–3916. 10.1097/00001756-200112210-00012 11742210

[B11] Bodineau L. Frugiere A. Marlot D. Wallois F. (2000b). Connections between retrotrapezoid nucleus and nucleus tractus solitarii in cat. Neurosci. Lett. 280, 111–114. 10.1016/S0304-3940(00)00770-9 10686390

[B12] Bodineau L. Taveau C. Le Quan Sang H. H. Osterstock G. Queguiner I. Moos F.. (2011). Data supporting a new physiological role for brain apelin in the regulation of hypothalamic oxytocin neurons in lactating rats. Endocrinology 152, 3492–3503. 10.1210/en.2011-0206 21733827

[B13] Breen S. Rees S. Walker D. (1997). Identification of brainstem neurons responding to hypoxia in fetal and newborn sheep. Brain Res. 748, 107–121. 10.1016/S0006-8993(96)01273-5 9067451

[B14] Bryan A. Bowas G. Maloney J. (1986). Control of breathing in the fetus and the newborn, in Handbook of Physiology, The Respiratory System, Control of Breathing, Vol 2, eds Cherniack N. S. Widdicombe J. G. (Washington, DC: American Physiological Society), 621–647.

[B15] Cao Y. Matsuyama K. Fujito Y. Aoki M. (2006). Involvement of medullary GABAergic and serotonergic raphe neurons in respiratory control: electrophysiological and immunohistochemical studies in rats. Neurosci. Res. 56, 322–331. 10.1016/j.neures.2006.08.001 16962678

[B16] Carroll J. L. Agarwal A. (2010). Development of ventilatory control in infants. Paediatr. Respir. Rev. 11, 199–207. 10.1016/j.prrv.2010.06.002 21109177

[B17] Danneman P. J. Mandrell T. D. (1997). Evaluation of five agents/methods for anesthesia of neonatal rats. Lab. Anim. Sci. 47, 386–395. 9306312

[B18] Darnall R. A. Schneider R. W. Tobia C. M. Commons K. G. (2016). Eliminating medullary 5-HT neurons delays arousal and decreases the respiratory response to repeated episodes of hypoxia in neonatal rat pups. J. Appl. Physiol. 120, 514–525. 10.1152/japplphysiol.00560.2014 26702023 PMC4773643

[B19] Dobbins E. G. Feldman J. L. (1995). Differential innervation of protruder and retractor muscles of the tongue in rat. J. Comp. Neurol. 357, 376–394. 10.1002/cne.903570305 7673474

[B20] Drorbaugh J. E. Fenn W. O. (1955). A barometric method for measuring ventilation in newborn infants. Pediatrics 16, 81–87. 14394741

[B21] Dubreuil V. Thoby-Brisson M. Rallu M. Persson K. Pattyn A. Birchmeier C.. (2009). Defective respiratory rhythmogenesis and loss of central chemosensitivity in Phox2b mutants targeting retrotrapezoid nucleus neurons. J. Neurosci. 29, 14836–14846. 10.1523/JNEUROSCI.2623-09.2009 19940179 PMC6665996

[B22] Enhorning G. Van S. S. Lundgren C. Vargas I. (1998). Whole-body plethysmography, does it measure tidal volume of small animals? Can. J. Physiol. Pharmacol. 76, 945–951. 10.1139/y99-002 10100875

[B23] Erickson J. T. Millhorn D. E. (1991). Fos-like protein is induced in neurons of the medulla oblongata after stimulation of the carotid sinus nerve in awake and anesthetized rats. Brain Res. 567, 11–24. 10.1016/0006-8993(91)91430-9 1815818

[B24] Erickson J. T. Millhorn D. E. (1994). Hypoxia and electrical stimulation of the carotid sinus nerve induce Fos-like immunoreactivity within catecholaminergic and serotoninergic neurons of the rat brainstem. J. Comp. Neurol. 348, 161–182. 10.1002/cne.903480202 7814687

[B25] Fay R. A. Norgren R. (1997). Identification of rat brainstem multisynaptic connections to the oral motor nuclei using pseudorabies virus. III. Lingual muscle motor systems. Brain Res. Brain Res. Rev. 25, 291–311. 10.1016/S0165-0173(97)00028-3 9495560

[B26] Fenik V. B. Rukhadze I. Kubin L. (2008). Inhibition of pontine noradrenergic A7 cells reduces hypoglossal nerve activity in rats. Neuroscience 157, 473–482. 10.1016/j.neuroscience.2008.08.069 18838113 PMC5222573

[B27] Filiano J. J. Kinney H. C. (1992). Arcuate nucleus hypoplasia in the sudden infant death syndrome. J. Neuropathol. Exp. Neurol. 51, 394–403. 10.1097/00005072-199207000-00002 1619439

[B28] Finley J. C. Katz D. M. (1992). The central organization of carotid body afferent projections to the brainstem of the rat. Brain Res. 572, 108–116. 10.1016/0006-8993(92)90458-L 1611506

[B29] Funk G. D. Zwicker J. D. Selvaratnam R. Robinson D. M. (2011). Noradrenergic modulation of hypoglossal motoneuron excitability: developmental and putative state-dependent mechanisms. Arch. Ital. Biol. 149, 426–453. 10.4449/aib.v149i4.1271 22205594

[B30] Gargaglioni L. H. Coimbra N. C. Branco L. G. (2003). The nucleus raphe magnus modulates hypoxia-induced hyperventilation but not anapyrexia in rats. Neurosci. Lett. 347, 121–125. 10.1016/S0304-3940(03)00671-2 12873742

[B31] Gauda E. B. (2006). Introduction: knowledge gained from animal studies of the fetus and newborn: application to the human premature infant. ILAR J. 47, 1–4. 10.1093/ilar.47.1.1

[B32] Gaultier C. Dauger S. Simonneau M. Gallego J. (2003). Genes modulating chemical breathing control: lessons from mutant animals. Respir. Physiol. Neurobiol. 136, 105–114. 10.1016/S1569-9048(03)00075-2 12853003

[B33] Gaultier C. Matrot B. Gallego J. (2006). Transgenic models to study disorders of respiratory control in newborn mice. ILAR J. 47, 15–21. 10.1093/ilar.47.1.15 16391427

[B34] Gluckman P. D. Johnston B. M. (1987). Lesions in the upper lateral pons abolish the hypoxic depression of breathing in unanaesthetized fetal lambs *in utero*. J. Physiol. 382, 373–383. 10.1113/jphysiol.1987.sp016372 3625554 PMC1183029

[B35] Gordon C. (1993). Temperature Regulation in Laboratory Rodents. New York, NY: Cambridge University Press.

[B36] Gozal D. Gaultier C. (2001). Evolving concepts of the maturation of central pathways underlying the hypoxic ventilatory response. Am. J. Respir. Crit. Care Med. 164, 325–329. 10.1164/ajrccm.164.2.2011133 11463609

[B37] Guyenet P. G. Bayliss D. A. (2015). Neural control of breathing and CO2 homeostasis. Neuron 87, 946–961. 10.1016/j.neuron.2015.08.001 26335642 PMC4559867

[B38] Guyenet P. G. Mulkey D. K. (2010). Retrotrapezoid nucleus and parafacial respiratory group. Respir. Physiol. Neurobiol. 173, 244–255. 10.1016/j.resp.2010.02.005 20188865 PMC2891992

[B39] Guyenet P. G. Mulkey D. K. Stornetta R. L. Bayliss D. A. (2005). Regulation of ventral surface chemoreceptors by the central respiratory pattern generator. J. Neurosci. 25, 8938–8947. 10.1523/JNEUROSCI.2415-05.2005 16192384 PMC6725580

[B40] Hayward L. F. Felder R. B. (1995). Peripheral chemoreceptor inputs to the parabrachial nucleus of the rat. Am. J. Physiol. 268, R707–R714. 7900914 10.1152/ajpregu.1995.268.3.R707

[B41] Herdegen T. Leah J. D. (1998). Inducible and constitutive transcription factors in the mammalian nervous system: control of gene expression by Jun, Fos and Krox, and CREB/ATF proteins. Brain Res. Brain Res. Rev. 28, 370–490. 10.1016/S0165-0173(98)00018-6 9858769

[B42] Herman J. K. O'halloran K. D. Mitchell G. S. Bisgard G. E. (1999). Methysergide augments the acute, but not the sustained, hypoxic ventilatory response in goats. Respir. Physiol. 118, 25–37. 10.1016/S0034-5687(99)00070-5 10568417

[B43] Hirooka Y. Polson J. W. Potts P. D. Dampney R. A. (1997). Hypoxia-induced Fos expression in neurons projecting to the pressor region in the rostral ventrolateral medulla. Neuroscience 80, 1209–1224. 10.1016/S0306-4522(97)00111-5 9284071

[B44] Holtman J. R. Jr. Marion L. J. Speck D. F. (1990). Origin of serotonin-containing projections to the ventral respiratory group in the rat. Neuroscience 37, 541–552. 10.1016/0306-4522(90)90422-Z 2133358

[B45] Horn E. M. Kramer J. M. Waldrop T. G. (2000). Development of hypoxia-induced Fos expression in rat caudal hypothalamic neurons. Neuroscience 99, 711–720. 10.1016/S0306-4522(00)00221-9 10974434

[B46] Huckstepp R. T. Cardoza K. P. Henderson L. E. Feldman J. L. (2015). Role of parafacial nuclei in control of breathing in adult rats. J. Neurosci. 35, 1052–1067. 10.1523/JNEUROSCI.2953-14.2015 25609622 PMC4300318

[B47] Hunt C. E. (2001). Sudden infant death syndrome and other causes of infant mortality: diagnosis, mechanisms, and risk for recurrence in siblings. Am. J. Respir. Crit. Care Med. 164, 346–357. 10.1164/ajrccm.164.3.9910045 11500332

[B48] Jansen A. S. Nguyen X. V. Karpitskiy V. Mettenleiter T. C. Loewy A. D. (1995). Central command neurons of the sympathetic nervous system: basis of the fight-or-flight response. Science 270, 644–646. 10.1126/science.270.5236.644 7570024

[B49] Kaur C. You Y. Singh J. Peng C. M. Ling E. A. (2001). Expression of Fos immunoreactivity in some catecholaminergic brainstem neurons in rats following high-altitude exposure. J. Neurosci. Res. 63, 54–63. 10.1002/1097-4547(20010101)63:1<54::AID-JNR7>3.0.CO;2-X 11169614

[B50] Kawai A. Okada Y. Muckenhoff K. Scheid P. (1995). Theophylline and hypoxic ventilatory response in the rat isolated brainstem-spinal cord. Respir. Physiol. 100, 25–32. 10.1016/0034-5687(94)00124-I 7604181

[B51] Kinney H. C. Broadbelt K. G. Haynes R. L. Rognum I. J. Paterson D. S. (2011). The serotonergic anatomy of the developing human medulla oblongata: implications for pediatric disorders of homeostasis. J. Chem. Neuroanat. 41, 182–199. 10.1016/j.jchemneu.2011.05.004 21640183 PMC3134154

[B52] Lavezzi A. M. (2015). A new theory to explain the underlying pathogenetic mechanism of sudden infant death syndrome. Front. Neurol. 6:220. 10.3389/fneur.2015.00220 26539157 PMC4610199

[B53] Lazarenko R. M. Milner T. A. Depuy S. D. Stornetta R. L. West G. H. Kievits J. A.. (2009). Acid sensitivity and ultrastructure of the retrotrapezoid nucleus in Phox2b-EGFP transgenic mice. J. Comp. Neurol. 517, 69–86. 10.1002/cne.22136 19711410 PMC2826801

[B54] Mallard C. Vexler Z. S. (2015). Modeling ischemia in the immature brain: how translational are animal models? Stroke 46, 3006–3011. 10.1161/STROKEAHA.115.007776 26272384 PMC4589478

[B55] Mathew O. P. (2011). Apnea of prematurity: pathogenesis and management strategies. J. Perinatol. 31, 302–310. 10.1038/jp.2010.126 21127467

[B56] Matrot B. Durand E. Dauger S. Vardon G. Gaultier C. Gallego J. (2005). Automatic classification of activity and apneas using whole body plethysmography in newborn mice. J. Appl. Physiol. 98, 365–370. 10.1152/japplphysiol.00803.2004 15591306

[B57] Menuet C. Kourdougli N. Hilaire G. Voituron N. (2011). Differences in serotoninergic metabolism possibly contribute to differences in breathing phenotype of FVB/N and C57BL/6J mice. J. Appl. Physiol. 110, 1572–1581. 10.1152/japplphysiol.00117.2011 21415169

[B58] Morris K. F. Baekey D. M. Nuding S. C. Dick T. E. Shannon R. Lindsey B. G. (2003). Invited review: neural network plasticity in respiratory control. J. Appl. Physiol. 94, 1242–1252. 10.1152/japplphysiol.00715.2002 12571145

[B59] Mortola J. P. (2004). Implications of hypoxic hypometabolism during mammalian ontogenesis. Respir. Physiol. Neurobiol. 141, 345–356. 10.1016/j.resp.2004.01.011 15288604

[B60] Mortola J. P. Frappell P. B. (1998). On the barometric method for measurements of ventilation, and its use in small animals. Can. J. Physiol. Pharmacol. 76, 937–944. 10.1139/y99-001 10100874

[B61] Nattie E. E. (2001). Central chemosensitivity, sleep, and wakefulness. Respir. Physiol. 129, 257–268. 10.1016/S0034-5687(01)00295-X 11738659

[B62] Neubauer J. A. Melton J. E. Edelman N. H. (1990). Modulation of respiration during brain hypoxia. J. Appl. Physiol. 68, 441–451. 2180894 10.1152/jappl.1990.68.2.441

[B63] Nitsos I. Walker D. W. (1999). Characterization of pontine neurons which respond to hypoxia in fetal sheep. Neurosci. Lett. 266, 33–36. 10.1016/S0304-3940(99)00249-9 10336177

[B64] Nolan P. C. Waldrop T. G. (1993). *In vivo* and *in vitro* responses of neurons in the ventrolateral medulla to hypoxia. Brain Res. 630, 101–114. 10.1016/0006-8993(93)90648-7 8118678

[B65] Nunez-Abades P. A. Morillo A. M. Pasaro R. (1993). Brainstem connections of the rat ventral respiratory subgroups: afferent projections. J. Auton. Nerv. Syst. 42, 99–118. 10.1016/0165-1838(93)90042-S 8383713

[B66] Okada Y. Chen Z. Jiang W. Kuwana S. Eldridge F. L. (2002). Anatomical arrangement of hypercapnia-activated cells in the superficial ventral medulla of rats. J. Appl. Physiol. 93, 427–439. 10.1152/japplphysiol.00620.2000 12133847

[B67] Onimaru H. Ikeda K. Kawakami K. (2008). CO2-sensitive preinspiratory neurons of the parafacial respiratory group express Phox2b in the neonatal rat. J. Neurosci. 28, 12845–12850. 10.1523/JNEUROSCI.3625-08.2008 19036978 PMC6671793

[B68] Paterson D. S. Trachtenberg F. L. Thompson E. G. Belliveau R. A. Beggs A. H. Darnall R.. (2006). Multiple serotonergic brainstem abnormalities in sudden infant death syndrome. JAMA 296, 2124–2132. 10.1001/jama.296.17.2124 17077377

[B69] Paxinos G. Franklin K. (2001). The Mouse Brain in Stereotaxic Coordinates. San Diego, CA: Academic Press.

[B70] Paxinos G. Halliday G. Watson C. Koutcherov Y. Wang H. (2007). Developing Mouse Brain. London: Elsevier Academic Press.

[B71] Perez H. Ruiz S. (1995). Medullary responses to chemoreceptor activation are inhibited by locus coeruleus and nucleus raphe magnus. Neuroreport 6, 1373–1376. 10.1097/00001756-199507100-00003 7488727

[B72] Perrin-Terrin A. S. Jeton F. Pichon A. Frugière A. Richalet J. P. Bodineau L. (2016). The c-FOS protein immunohistological detection: a useful tool as a marker of central pathways involved in specific physiological responses *in vivo* and *ex vivo*. J. Vis. Exp. 110:e53613 10.3791/53613 PMC4941991 27167092

[B73] Poets C. F. Meny R. G. Chobanian M. R. Bonofiglo R. E. (1999). Gasping and other cardiorespiratory patterns during sudden infant deaths. Pediatr. Res. 45, 350–354. 10.1203/00006450-199903000-00010 10088653

[B74] Richter D. W. Schmidt-Garcon P. Pierrefiche O. Bischoff A. M. Lalley P. M. (1999). Neurotransmitters and neuromodulators controlling the hypoxic respiratory response in anaesthetized cats. J. Physiol. 514(Pt 2), 567–578. 10.1111/j.1469-7793.1999.567ae.x 9852336 PMC2269078

[B75] Rose M. F. Ren J. Ahmad K. A. Chao H. T. Klisch T. J. Flora A.. (2009). Math1 is essential for the development of hindbrain neurons critical for perinatal breathing. Neuron 64, 341–354. 10.1016/j.neuron.2009.10.023 19914183 PMC2818435

[B76] Ruffault P. L. D'Autréaux F. Hayes J. A. Nomaksteinsky M. Autran S. Fujiyama T. (2015). The retrotrapezoid nucleus neurons expressing *Atoh1* and *Phox2b* are essential for the respiratory response to CO_2_. Elife 4:e07051 10.7554/eLife.07051 PMC4429526 25866925

[B77] Runold M. Lagercrantz H. Prabhakar N. R. Fredholm B. B. (1989). Role of adenosine in hypoxic ventilatory depression. J. Appl. Physiol. 67, 541–546. 2793655 10.1152/jappl.1989.67.2.541

[B78] Simakajornboon N. Vlasic V. Li H. Sawnani H. (2004). Effect of prenatal nicotine exposure on biphasic hypoxic ventilatory response and protein kinase C expression in caudal brain stem of developing rats. J. Appl. Physiol. 96, 2213–2219. 10.1152/japplphysiol.00935.2003 14752122

[B79] Soulier V. Gestreau C. Borghini N. Dalmaz Y. Cottet-Emard J. M. Pequignot J. M. (1997). Peripheral chemosensitivity and central integration: neuroplasticity of catecholaminergic cells under hypoxia. Comp. Biochem. Physiol. A Physiol. 118, 1–7. 10.1016/S0300-9629(96)00369-6 9243809

[B80] Stornetta R. L. Guyenet P. G. (1999). Distribution of glutamic acid decarboxylase mRNA-containing neurons in rat medulla projecting to thoracic spinal cord in relation to monoaminergic brainstem neurons. J. Comp. Neurol. 407, 367–380. 10.1002/(SICI)1096-9861(19990510)407:3<367::AID-CNE5>3.0.CO;2-6 10320217

[B81] Stornetta R. L. Moreira T. S. Takakura A. C. Kang B. J. Chang D. A. West G. H.. (2006). Expression of Phox2b by brainstem neurons involved in chemosensory integration in the adult rat. J. Neurosci. 26, 10305–10314. 10.1523/JNEUROSCI.2917-06.2006 17021186 PMC6674621

[B82] Stornetta R. L. Rosin D. L. Simmons J. R. McQuiston T. J. Vujovic N. Weston M. C.. (2005). Coexpression of vesicular glutamate transporter-3 and gamma-aminobutyric acidergic markers in rat rostral medullary raphe and intermediolateral cell column. J. Comp. Neurol. 492, 477–494. 10.1002/cne.20742 16228993

[B83] Takakura A. C. Moreira T. S. Colombari E. West G. H. Stornetta R. L. Guyenet P. G. (2006). Peripheral chemoreceptor inputs to retrotrapezoid nucleus (RTN) CO2-sensitive neurons in rats. J. Physiol. 572, 503–523. 10.1113/jphysiol.2005.103788 16455687 PMC1779666

[B84] Teppema L. J. Dahan A. (2010). The ventilatory response to hypoxia in mammals: mechanisms, measurement, and analysis. Physiol. Rev. 90, 675–754. 10.1152/physrev.00012.2009 20393196

[B85] Teppema L. J. Veening J. G. Kranenburg A. Dahan A. Berkenbosch A. Olievier C. (1997). Expression of c-fos in the rat brainstem after exposure to hypoxia and to normoxic and hyperoxic hypercapnia. J. Comp. Neurol. 388, 169–190. 10.1002/(SICI)1096-9861(19971117)388:2<169::AID-CNE1>3.0.CO;2-# 9368836

[B86] Tree K. C. Scotto Di Perretolo M. Peyronnet J. Cayetanot F. (2014). *In utero* cannabinoid exposure alters breathing and the response to hypoxia in newborn mice. Eur. J. Neurosci. 40, 2196–2204. 10.1111/ejn.12588 24717006

[B87] Vizek M. Pickett C. K. Weil J. V. (1987). Biphasic ventilatory response of adult cats to sustained hypoxia has central origin. J. Appl. Physiol. 63, 1658–1664. 3693202 10.1152/jappl.1987.63.4.1658

[B88] Voituron N. Frugiere A. Champagnat J. Bodineau L. (2006). Hypoxia-sensing properties of the newborn rat ventral medullary surface *in vitro*. J. Physiol. 577, 55–68. 10.1113/jphysiol.2006.111765 16901937 PMC2000692

[B89] Voituron N. Frugiere A. Mc Kay L. C. Romero-Granados R. Dominguez-Del-Toro E. Saadani-Makki F.. (2011). The kreisler mutation leads to the loss of intrinsically hypoxia-activated spots in the region of the retrotrapezoid nucleus/parafacial respiratory group. Neuroscience 194, 95–111. 10.1016/j.neuroscience.2011.07.062 21839147

[B90] Waldrop T. G. Porter J. P. (1995). Hypothalamic involvement in respiratory and cardiovascular regulation, in Regulation of Breathing, ed Dempsey J. A. (New York, NY: Marcel Dekker), 315–364.

[B91] Walker D. W. (1995). Hypoxic inhibition of breathing and motor activity in the foetus and newborn. Clin. Exp. Pharmacol. Physiol. 22, 533–536. 10.1111/j.1440-1681.1995.tb02062.x 7586709

[B92] Walker D. W. Lee B. Nitsos I. (2000). Effect of hypoxia on respiratory activity in the foetus. Clin. Exp. Pharmacol. Physiol. 27, 110–113. 10.1046/j.1440-1681.2000.03201.x 10696538

[B93] Weston M. C. Stornetta R. L. Guyenet P. G. (2004). Glutamatergic neuronal projections from the marginal layer of the rostral ventral medulla to the respiratory centers in rats. J. Comp. Neurol. 473, 73–85. 10.1002/cne.20076 15067719

